# Structure–Function Decoupling of the Sensorimotor and Default Mode Networks in Black Americans With MS

**DOI:** 10.1002/acn3.70331

**Published:** 2026-02-12

**Authors:** Emilio Cipriano, Giacomo Boffa, Maria Petracca, Marta Ponzano, Nicole Graziano, Claire Wigley, Claire Riley, Jonathan Howard, Pietro Bontempi, Sylvia Klineova, Fred Lublin, Matilde Inglese

**Affiliations:** ^1^ Department of Neuroscience, Rehabilitation, Ophthalmology, Genetics, Maternal and Child Health (DINOGMI) University of Genoa Genoa Italy; ^2^ IRCCS Ospedale Policlinico San Martino Genoa Italy; ^3^ Department of Human Neurosciences Sapienza University of Rome Rome Italy; ^4^ Department of Health Sciences (DISSAL) University of Genoa Genoa Italy; ^5^ The Corinne Goldsmith Dickinson Center for Multiple Sclerosis Icahn School of Medicine at Mount Sinai New York New York USA; ^6^ Department of Neurology Columbia University Medical Center New York New York USA; ^7^ Department of Neurology New York University School of Medicine New York New York USA; ^8^ Department of Engineering for Innovation Medicine University of Verona Verona Italy

**Keywords:** functional connectivity, multiple sclerosis, structural connectivity, structure‐function coupling

## Abstract

**Background and Objectives:**

Multiple sclerosis (MS) exhibits racially disparate rates of disease progression. Black people with MS (B‐PwMS) experience a more severe disease course than non‐Hispanic White people with MS (NHW‐PwMS). Here we investigated structural and functional connectivity as well as structure–function decoupling in the sensorimotor and default mode networks (SMN and DMN, respectively), which play a key role in determining physical and cognitive disability in people with MS.

**Methods:**

A total of 176 participants (50 B‐PwMS, 50 NHW‐PwMS, 41 Black healthy controls (B‐HCs), and 35 NHW‐HCs) underwent 3T‐MRI with T1, resting‐state functional, & diffusion imaging, and clinical assessment with Expanded‐Disability‐Status‐Scale (EDSS) & Symbol‐Digit‐Modalities‐Test (SDMT). T1‐weighted images were lesion‐filled and segmented to obtain cortical, subcortical, and cerebellar structures. Diffusion and functional MRI datasets were preprocessed, and structural and functional connectivity were extracted between regions defined by the AAL3 atlas. Global and local network measures were extracted for both structural and functional connectivity, and structure–function decoupling was quantified by calculating the correlation between the strengths of the two networks, considering only edges with non‐zero structural connectivity. Network measures were compared between subgroups, accounting for the impact of demographics and social determinants of health, with correction for multiple comparisons.

**Results:**

Despite similar disease duration, treatment exposure, lesion load and brain volumes, B‐PwMS exhibited higher EDSS and lower SDMT scores compared to NHW‐PwMS, which were influenced by total income and body mass index. In both B‐ and NHW‐PwMS, structural global efficiency and streamline density were lower in the SMN and DMN compared to their respective HCs. Structural characteristic path length in SMN was significantly higher in B‐PwMS versus B‐HCs, whereas no significant differences were observed in NHW groups. Extensive local rearrangements of structural and functional hubs were observed in the SMN and DMN of B‐PwMS compared to B‐HCs and NHW‐PwMS. B‐PwMS showed greater structure–function decoupling in the SMN as compared to the other groups. There was a trend towards a higher decoupling in the DMN with lower SDMT scores (*ρ* = −0.20, *p* = 0.05), and higher decoupling in the SMN with higher EDSS scores (*ρ* = 0.20, *p* = 0.06).

**Discussion:**

Despite similar brain volumes and lesion load, B‐PwMS showed a greater rearrangement of brain structural and functional connectivity within the SMN and DMN when compared with NHW‐PwMS. Moreover, B‐PwMS showed a higher degree of structure–function decoupling in the SMN, with a trend towards association with increased physical disability.

## 1 Introduction

Racial disparities in the prevalence, progression, and clinical outcomes of persons with multiple sclerosis (PwMS) are well‐documented [1, 2]. Black people with MS (B‐PwMS) experience higher levels of disability, faster disease progression, and an overall more severe disease course when compared to non‐Hispanic White (NHW) PwMS [3, 4, 5]. These disparities arise from a complex interplay of genetic, immunological, and environmental factors, including socioeconomic status, healthcare access, and other social determinants of health (SDoH) [6, 7, 8]. Several studies have indeed shown that B‐PwMS have increased serum plasmablasts [9], neuron‐binding antibodies [10], and a higher IgG—the latter having been posited as a risk factor for cerebral gray matter atrophy [11]. MRI studies comparing B‐PwMS and NHW‐PwMS have demonstrated differences in lesion volume [12, 13], gray matter atrophy [14, 15], microstructural damage [13], and chronic inflammation [13]. Moreover, social adversity has a pervasive impact on brain structure development [16], on allostatic load [17], and on epigenetic modifications that may influence disease susceptibility and progression [18].

Tissue damage accrual affects the brain's structural and functional organization [19], potentially resulting in a disconnection syndrome characterized by neural connectivity disruptions and network dysfunction. Thus, by understanding the different adaptive and/or maladaptive mechanisms between B‐PwMS and NHW‐PwMS we may elucidate the mechanisms underlying this health disparity. Large‐scale brain networks including the frontoparietal, salience, sensorimotor (SMN), and default mode (DMN) systems have all been implicated in MS and contribute to cognitive and motor outcomes. We focused on the SMN and DMN for two complementary reasons. First, the SMN is directly linked to motor function and physical disability measures commonly used in MS and is particularly sensitive to white matter damage affecting long‐range sensorimotor pathways [20]. Second, the DMN is a well‐established substrate of processing speed [21] and has been repeatedly associated with cognitive impairment in MS [22]. Here, we employed an advanced MRI protocol to investigate differences in SMN and DMN structural and functional connectivity, as well as structure–function decoupling, between B‐PwMS, NHW‐PwMS, and their healthy counterparts. Structural connectivity (SC) reflects the anatomical architecture of the brain, capturing the integrity and organization of white matter pathways derived from diffusion MRI. Functional connectivity (FC), in contrast, represents the temporal coherence of neural activity between spatially distributed regions measured through resting‐state fMRI. Although SC provides information about the physical substrate that enables communication between brain areas, FC reflects the temporal coordination of neural activity between regions, which is shaped, but not fully determined, by the underlying structural architecture. Structure–function decoupling was included to capture potential mismatches between structural damage and functional network organization, which may reflect compensatory or maladaptive mechanisms and provide greater sensitivity to network dysfunction not detectable through structural or functional connectivity alone.

## 2 Methods

### 2.1 Study Population and Clinical Assessment

As previously reported in Petracca et al. [5], 120 PwMS and 82 HCs were prospectively enrolled; of these, 50 B‐PwMS, 50 NHW‐PwMS, 41 B‐HCs, and 35 NHW‐HCs underwent 3T brain MRI and were included in the present study. Subgroups were stratified by self‐reported race and matched for age and sex; all PwMS had a relapsing–remitting phenotype (RRMS). We collected data about total income (total combined family income for the past 12 months, nine ordinal variables categorized from <$5000 to $100,000+), years of education, and body mass index (BMI). All subjects underwent neurological examination with Expanded‐Disability‐Status‐Scale (EDSS) scoring and neuropsychological assessment with Symbol‐Digit‐Modalities‐Test (SDMT).

### 2.2 Standard Protocol Approvals, Registrations, and Patient Consents

Written informed consent was obtained from all participants before the beginning of the study procedures, according to the Declaration of Helsinki. The protocol was approved by the Icahn School of Medicine at Mount Sinai Institutional Review Board (17‐00766).

### 2.3 MRI Imaging Protocol

MRI data were acquired on a Siemens Skyra 3T scanner (Siemens, Erlangen, Germany) with a 32‐channel head coil. The protocol included anatomical imaging, resting‐state fMRI (rs‐fMRI) for functional connectivity analysis, and diffusion MRI (dMRI) for mapping WM tracts. The specific acquisition protocol consisted of: 3D‐MPRAGE (TR = 2400 ms, TE = 2 ms, TI = 1000 ms, voxel size = 1 × 1 × 1 mm^3^); 3D‐FLAIR (TR = 3200 ms, TE = 564 ms, voxel size = 1 × 1 × 1 mm^3^); GRE‐EPI sequence for rs‐fMRI (TR = 1000 ms, TE = 31.4 ms, Time point = 600, flip angle = 60°, voxel size = 2.3 × 2.3 × 2.3 mm^3^); twice‐refocused SE‐EPI for dMRI (TR = 4700 ms, TE = 100 ms, flip angle = 80°, voxel size = 1.8 × 1.8 × 1.8 mm^3^, with 30 directions each, repeated twice in left–right and right–left encoding, at *b* = 1000s/mm^2^ and b = 2000s/mm^2^ and *b* = 0 s/mm^2^).

### 2.4 Brain Network Construction

#### 2.4.1 Structural MRI Processing

Three PwMS were excluded from the analyses due to poor‐quality MRI scans. T2 hyperintense and T1 hypointense lesion volumes were identified and measured on 3D‐FLAIR and 3D‐MPRAGE images (Jim version 7.0; Xinapse Systems Ltd., West Bergholt, UK). The corresponding T1 images were then filled using T1‐hypointense lesion mask and FMRIB software library (FSL) [23]. Total brain volumes, cortical gray matter (GM), subcortical GM, and cerebellar volumes (normalized by total intracranial volume‐TIV‐), were obtained by segmenting the T1‐filled image using FreeSurfer (version 7.4.0) [24]. All FreeSurfer segmentations underwent visual quality control by an expert rater with over 5 years of experience in neuroimaging (GB). A total of 166 cortical and subcortical areas were extracted according to the third edition of the automated anatomical atlas (AAL3) (https://www.oxcns.org/aal3.html) and used as nodes for structural and functional connectivity networks. For this purpose, T1 filled images were registered to the MNI space using affine and non‐linear transformations in ANTs, and were also co‐registered with diffusion and functional images using rigid transformations in ANTs [25].

#### 2.4.2 Functional MRI Processing

Functional data preprocessing was performed using the FSL's tool FEAT. The pipeline included realignment, slice timing correction, smoothing, and denoising. FC network was then extracted for each subject using AFNI (https://afni.nimh.gov/). Nodes for the FC network were derived from the cortical parcellation based on the AAL3 atlas. The SMN included the following regions of interest: bilateral pre‐ and post‐central cortex, supplementary motor cortex, thalamus, caudate, putamen, pallidum, and cerebellum (vermis & cerebellar hemispheres); the DMN included: the cingulate, cuneus, precuneus, inferior/middle/superior/orbito‐frontal, parahippocampal, middle/superior temporal, and angular & supramarginal cortices (Table S1). Links for the FC network were computed as the Pearson correlation coefficient between the time series of each pair of nodes. To reduce noise‐driven weak correlations and obtain a meaningful graph representation, the resulting FC matrices were thresholded and binarized. Thresholding was performed using the Orthogonal Minimal Spanning Trees (OMST) approach, a data‐driven method that identifies the optimal set of connections by maximizing the balance between global efficiency and wiring cost [26]. This procedure avoids arbitrary threshold selection and yields a sparse, biologically meaningful, and cross‐subject–comparable network structure.

#### 2.4.3 Diffusion MRI Processing

Diffusion MR images underwent de‐noising using the Marchenko‐Pastur principal component analysis algorithm in MRtrix3 [27]; motion artifacts and susceptibility‐induced distortions were corrected using FSL's eddy and top‐up commands. B1 field inhomogeneity correction was applied to dMRIs using ANTs N4 algorithm.

Fiber tractography (MRtrix3 package) was performed using constrained spherical deconvolution (CSD) with a maximum number of streamlines of 2 million via the iFOD2 algorithm, which facilitates more accurate fiber reconstruction in heavily curved regions. The Anatomically‐Constrained Tractography (ACT) [27] option was applied using 5‐tissue‐type (5TT) segmented images to improve tractography accuracy and enhance the anatomical plausibility of the reconstructed fibers, discarding anatomically unfeasible streamlines. Previously identified lesion maps were also incorporated as the optional 5th tissue type in the 5TT maps to prevent tissue‐type misclassifications due to T2 hyperintensities [28]. Additionally, Spherical‐deconvolution Informed Filtering of Tractograms (SIFT) algorithms were applied to remove streamlines unlikely to represent true anatomical connections while preserving those that are more biologically plausible [27]. Using the obtained tractography, the SC network was extracted for each subject using the same nodes as those used for the FC network, based on the AAL3 atlas. Links for the SC network were computed as the number of streamlines between two different nodes.

#### 2.4.4 Network Measures

Global and local graph measures for both structural and functional networks were extracted in the SMN and DMN using the Brain Connectivity Toolbox (BCT, https://github.com/aestrivex/bctpy). Global graph measures provide information about the overall structure of the graph; local graph measures offer insight into individual nodes within the graph while helping to identify influential nodes within the network. Specifically, we computed global network metrics including density, global efficiency, clustering coefficient, and characteristic path length. Density quantifies the proportion of existing connections relative to all possible connections in the network, providing a measure of overall network connectivity. Global efficiency reflects the ability of the network to integrate information across distant regions, indicating how efficiently information can be exchanged at the global level. Clustering coefficient measures the tendency of nodes to form tightly interconnected clusters, capturing local segregation and modular organization. Characteristic path length represents the average shortest path between all pairs of nodes, providing a complementary measure of network integration and communication efficiency.

Local measures included node‐wise degree centrality and participation coefficient (PC). Degree centrality identifies nodes with a high number of connections, highlighting key regions that may influence information flow within the network. The participation coefficient quantifies how a node's connections are distributed across different modules, distinguishing nodes that link multiple clusters (connector hubs) from those primarily connected within their own cluster (provincial hubs) [29]. Nodes with a degree centrality values at least one standard deviation above the mean were classified as hubs [30]—highly connected nodes that play a crucial role in clusters, by reinforcing connectivity within their own cluster or by linking different clusters together. We further categorized these hubs as inter‐cluster (connector) or intra‐cluster (provincial) hubs based on their PC. A high PC indicated a connector hub, and low PCs indicated provincial hubs; specifically, hubs were classified as provincial if they exceeded the third quartile of the degree distribution.

#### 2.4.5 Structure–Function Decoupling

We evaluated structure–function connectivity coupling by calculating the correlation coefficient between the strengths of the two networks, limited to edges with non‐zero structural connectivity [31]. Edges with non‐zero structural connectivity were extracted to create a vector of structural connectivity values, which were then rescaled to follow a Gaussian distribution; corresponding vectors of functional connectivity were also extracted. Finally, the Pearson's correlation coefficient was computed using the resultant vectors. This coupling was assessed both within the SMN and DMN.

**FIGURE 1:**
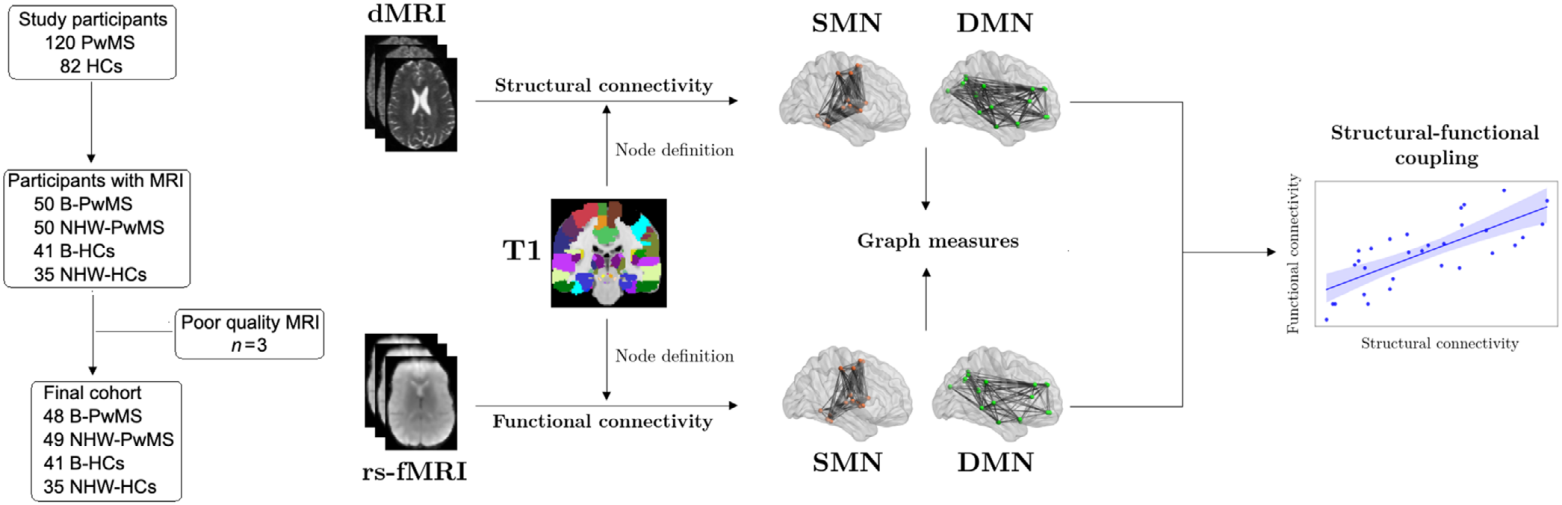
Flow‐chart diagram showing the patient cohort and the analysis pipeline of the study. B‐HCs, Black healthy controls; B‐PwMS, Black people with Multiple Sclerosis; DMN, Default mode network; dMRI, diffusion MRI; HCs, Healthy controls; NWH‐HCs, Non‐Hispanic White healthy controls; NWH‐PwMS, Non‐Hispanic White people with Multiple Sclerosis; PwMS, People with Multiple Sclerosis; rs‐fMRI, resting state functional MRI; SMN, Sensorimotor network.

Figure 1 summarizes the steps of the data analysis pipeline.

### 2.5 Statistical Analysis

Statistical analyses were performed using Python (v.3.9.19). Descriptive statistics were reported as mean with standard deviation (SD), median with interquartile range (IQR), or range, based on variable distribution. Group comparisons of demographic, clinical variables, SDoH and basic MRI measures across all four groups (B‐HCs, B‐PwMS, NHW‐HCs, NHW‐PwMS) were first performed using ANOVA, Kruskal–Wallis or chi‐square tests, as appropriate. Post hoc comparisons between study subgroups (B‐PwMS vs. B‐HCs, NHW‐PwMS vs. NHW‐HCs, and B‐PwMS vs. NHW‐PwMS) were then performed for variables showing significant group effects, with adjustment for age, sex, years of education, total income and BMI using general linear models (GLMs). For graph measures of SC, FC, and SC–FC coupling, *p*‐values were initially calculated across all four groups, and false discovery rate (FDR) correction was applied across these tests to identify statistically significant differences. Metrics that survived FDR correction were subsequently compared between study subgroups. Finally, significant graph and coupling measures were correlated with clinical variables using Spearman correlation analysis. Two‐tailed *p*‐values < 0.05 were considered statistically significant.

## 3 Results

Table 1 shows the clinical and MRI characteristics of the study population. No differences were noted in disease duration, treatment exposure, lesion load, and brain volumes between B‐PwMS and NHW‐PwMS. B‐PwMS had lower total incomes, higher BMI, and slightly lower years of education than NHW‐PwMS and exhibited higher EDSS scores and lower SDMT scores. EDSS, SDMT, and brain volumes were influenced by BMI and total income (Table S2). After adjustment for these covariates, differences in EDSS and SDMT scores between B‐PwMS and NHW‐PwMS were not statistically significant.

**TABLE 1:** Demographics and MRI characteristics in Black and Non‐Hispanic white patients with multiple sclerosis compared to their healthy controls' counterparts.

	B‐HCs	B‐PwMS	NHW‐HCs	NHW‐PwMS	*p*	Post hoc *p*
Female, number (%)	29 (71)	35 (73)	22 (63)	31 (63)	0.54	—
Age, years, mean (SD)	36.4 (11.9)	37.7 (10.4)	33.4 (9.9)	36.0 (7.8)	0.49	—
Disease duration, years, mean (SD)	—	5.8 (5.8)	—	5.8 (4.9)	0.36	—
Treatment, *n* (%)	—		—		0.5	—
None		5 (10)		2 (4)		
Low/moderate efficacy		19 (40)		21 (43)		
High efficacy		24 (50)		26 (53)		
EDSS, median (IQR)	—	2.0 (1.0–3.1)	—	1.0 (0.0–2.0)	0.03	—
SDMT, mean (SD)	56.9 (13.4)	51.9 (10.3)	67.3 (9.31)	57.9 (9.37)	< 0.001	B‐HCs vs. B‐PwMS: 0.061 NHW‐HCs vs. NHW‐PwMS: < 0.001 B‐PwMS vs. NHW‐PwMS: < 0.001
Total income, median (IQR)	6.0 (4.0–7.0)	4.5 (3.7–6.2)	7.0 (6.0–7.0)	6.0 (5.0–8.0)	0.005	B‐HCs vs. B‐PwMS: 0.431 NHW‐HCs vs. NHW‐PwMS: 0.599 B‐PwMS vs. NHW‐PwMS: 0.019
Education, mean (SD)	15.5 (2.4)	15.9 (1.8)	19.1 (3.7)	17.5 (3.2)	< 0.001	B‐HCs vs. B‐PwMS: 0.923 NHW‐HCs vs. NHW‐PwMS: 0.026 B‐PwMS vs. NHW‐PwMS: 0.001
Body mass index	27.6 (4.0)	30.2 (5.2)	23.6 (4.5)	26.3 (5.6)	< 0.001	B‐HCs vs. B‐PwMS: 0.037 NHW‐ HCs vs. NHW‐PwMS: < 0.001 B‐PwMS vs. NHW‐PwMS: 0.037
Normalized total brain volume, mean (SD)	0.73 (0.03)	0.72 (0.04)	0.70 (0.03)	0.71 (0.03)	0.002	B‐HCs vs. B‐PwMS: 0.008 NHW‐ HCs vs. NHW‐PwMS: 0.180 B‐PwMS vs. NHW‐PwMS: 0.609
Normalized WM volume, mean (SD)	0.29 (0.02)	0.27 (0.03)	0.29 (0.02)	0.28 (0.02)	0.007	B‐HCs vs. B‐PwMS: 0.007 NHW‐HCs vs. NHW‐PwMS: 0.355 B‐PwMS vs. NHW‐PwMS: 0.411
Normalized cortical GM volume, mean (SD)	0.30 (0.02)	0.29 (0.02)	0.30 (0.01)	0.29 (0.01)	0.005	B‐HCs vs. B‐PwMS: 0.183 NHW‐HCs vs. NHW‐PwMS: 0.144 B‐PwMS vs. NHW‐PwMS: 0.361
FLAIR lesion volume, mL, mean (SD)	—	4.32 (5.19)	—	3.95 (5.78)	0.79	—

*Note:* *p*‐values refer to Anova, Kruskal–Wallis or chi‐square tests as appropriate.

Abbreviations: B‐HCs, Black healthy controls; B‐PwMS, Black people with Multiple Sclerosis; EDSS, Expanded‐Disability‐Status‐Scale; GM, Gray matter; IQR, interquartile range; NWH‐HCs, Non‐Hispanic White healthy controls; NWH‐PwMS, Non‐Hispanic White people with Multiple Sclerosis; SD, standard deviation; SDMT, Symbol‐Digit‐Modalities‐Test; WM, White Matter.

### 3.1 Structural Connectivity Measures

As shown in Table 2, several global structural connectivity measures differed significantly between study subgroups. After correction for multiple comparisons and adjustment for age, sex, BMI, years of education and total income, both subgroups of PwMS showed significantly lower global efficiency as well as reduced streamline density in SMN and DMN, compared to their respective HCs (Table 3). Additionally, characteristic path length in the SMN was significantly increased in B‐PwMS vs B‐HCs, whereas no significant differences were observed in NHW subgroups. Higher global efficiency (*ρ* = −0.31, *p* = 0.002), clustering coefficient (*ρ* = −0.24, *p* = 0.02), and streamline density (*ρ* = −0.32, *p* = 0.002) within the SMN were associated with lower EDSS scores across both patient groups.

**TABLE 2:** Graph measures of structural connectivity (SC), functional connectivity (FC), and SC–FC coupling.

	B‐HCs	B‐PwMS	NHW‐HCs	NHW‐PwMs	*p*
Structural connectivity					
Global efficiency: SMN	0.92 (0.91; 0.93)	0.91 (0.90; 0.92)	0.92 (0.91; 0.93)	0.91 (0.90; 0.92)	0.0004
Global efficiency: DMN	0.84 (0.83; 0.85)	0.82 (0.79; 0.85)	0.84 (0.82; 0.85)	0.81 (0.79; 0.83)	0.0002
Clustering coefficient: SMN	0.10 (0.08; 0.12)	0.09 (0.07; 0.11)	0.09 (0.08; 0.10)	0.08 (0.07; 0.11)	0.077
Clustering coefficient: DMN	0.19 (0.16; 0.23)	0.19 (0.15; 0.23)	0.20 (0.18; 0.22)	0.19 (0.15; 0.22)	0.503
Characteristic path length: SMN	2.24 (2.13; 2.40)	2.38 (2.24; 2.61)	2.33 (2.18; 2.51)	2.35 (2.23; 2.52)	0.002
Characteristic path length: DMN	2.93 (2.61; 3.23)	2.84 (2.54; 3.16)	2.90 (2.65; 3.20)	2.77 (2.55; 3.15)	0.827
Density: SMN	0.84 (0.82; 0.86)	0.81 (0.79; 0.84)	0.84 (0.81; 0.85)	0.82 (0.79; 0.84)	0.0004
Density: DMN	0.68 (0.66; 0.69)	0.65 (0.59; 0.69)	0.67 (0.63; 0.70)	0.63 (0.59; 0.67)	0.0002
Functional connectivity					
Global efficiency: SMN	0.23 (0.17; 0.32)	0.27 (0.20; 0.36)	0.22 (0.15; 0.30)	0.22 (0.17; 0.30)	0.073
Global efficiency: DMN	0.33 (0.26; 0.40)	0.33 (0.27; 0.36)	0.31 (0.26; 0.37)	0.32 (0.25; 0.37)	0.566
Clustering coefficient: SMN	0.33 (0.29; 0.43)	0.33 (0.26; 0.44)	0.33 (0.25; 0.39)	0.32 (0.25; 0.42)	0.345
Clustering coefficient: DMN	0.40 (0.35; 0.46)	0.41 (0.36; 0.48)	0.37 (0.29; 0.46)	0.39 (0.33; 0.44)	0.416
Characteristic path length: SMN	2.83 (2.19; 3.34)	3.03 (2.44; 3.51)	2.69 (2.10; 3.51)	2.80 (2.18; 3.20)	0.691
Characteristic path length: DMN	3.07 (2.71; 3.39)	2.94 (2.66; 3.50)	3.13 (2.57; 3.56)	3.07 (2.67; 3.44)	0.988
Density: SMN	0.11 (0.10; 0.12)	0.12 (0.10; 0.13)	0.10 (0.09; 0.12)	0.11 (0.09; 0.13)	0.074
Density: DMN	0.13 (0.12; 0.14)	0.13 (0.11; 0.14)	0.12 (0.11; 0.14)	0.13 (0.11; 0.14)	0.437
Structural‐functional coupling					
SMN	0.38 (0.36; 0.41)	0.35 (0.33; 0.38)	0.39 (0.36; 0.44)	0.39 (0.35; 0.43)	0.010
DMN	0.30 (0.28; 0.33)	0.29 (0.25; 0.32)	0.32 (0.27; 0.35)	0.31 (0.29; 0.34)	0.186

*Note:* Results are reported as median (IQR). *p*‐values refer to ANOVA or Kruskal–Wallis test as appropriate; all statistically significant comparisons were confirmed after adjustment for multiple comparisons using the false discovery rate (FDR) method.

Abbreviations: B‐HCs, Black healthy controls; B‐PwMS, Black people with Multiple Sclerosis; DMN, default mode network; NWH‐HCs, Non‐Hispanic White healthy controls; NWH‐PwMS, Non‐Hispanic White people with Multiple Sclerosis; SMN, sensorimotor network.

**TABLE 3:** Global structural connectivity (SC) metrics that survived group comparisons and correction for multiple comparisons using the false discovery rate method.

	Unadjusted		Adjusted	
	*β* (95% CI)	*p*	*β* (95% CI)	*p*
GE (SMN, SC)				
B‐HCs vs. B‐PwMS	0.01 (0.00; 0.02)	0.001	0.01 (0.00; 0.02)	0.002
NHW‐ HCs vs. NHW‐PwMS	0.01 (0.00; 0.02)	0.013	0.01 (0.00; 0.02)	0.012
B‐PwMS vs. NHW‐PwMS	−0.00 (−0.01; 0.01)	0.959	0.00 (−0.01; 0.01)	0.894
GE (DMN, SC)				
B‐HCs vs. B‐PwMS	0.02 (0.00; 0.03)	0.006	0.02 (0.01; 0.03)	0.003
NHW‐ HCs vs. NHW‐PwMS	0.02 (0.01; 0.03)	0.002	0.02 (0.01; 0.03)	0.001
B‐PwMS vs. NHW‐PwMS	0.01 (−0.00; 0.02)	0.212	0.01 (−0.01; 0.02)	0.307
CPL (SMN, SC)				
B‐HCs vs. B‐PwMS	−0.22 (−0.33; −0.11)	< 0.001	−0.21 (−0.32; −0.10)	< 0.001
NHW‐ HCs vs. NHW‐PwMS	−0.02 (−0.14; 0.09)	0.710	0.02 (−0.10; 0.13)	0.762
B‐PwMS vs. NHW‐PwMS	0.09 (−0.02; 0.19)	0.107	0.04 (−0.08; 0.15)	0.504
Density (SMN, SC)				
B‐HCs vs. B‐PwMS	0.02 (0.01; 0.04)	0.001	0.02 (0.01; 0.04)	0.002
NHW‐ HCs vs. NHW‐PwMS	0.02 (0.00; 0.03)	0.012	0.02 (0.00; 0.04)	0.012
B‐PwMS vs. NHW‐PwMS	−0.00 (−0.01; 0.01)	0.954	0.00 (−0.01; 0.02)	0.898
Density (DMN, SC)				
B‐HCs vs. B‐PwMS	0.03 (0.01; 0.06)	0.006	0.04 (0.01; 0.06)	0.003
NHW‐ HCs vs. NHW‐PwMS	0.04 (0.01; 0.06)	0.002	0.04 (0.02; 0.07)	0.001
B‐PwMS vs. NHW‐PwMS	0.01 (−0.01; 0.04)	0.200	0.01 (−0.01; 0.04)	0.290
SC‐FC coupling (SMN)				
B‐HCs vs. B‐PwMS	0.03 (0.00; 0.05)	0.035	0.03 (0.00; 0.06)	0.021
NHW‐ HCs vs. NHW‐PwMS	0.00 (−0.02; 0.03)	0.702	0.01 (−0.02; 0.03)	0.622
B‐PwMS vs. NHW‐PwMS	−0.03 (−0.06; −0.01)	0.005	−0.04 (−0.06; −0.01)	0.004

*Note:* Reported are estimates (*β*), 95% confidence intervals (CI), and *p*‐values from unadjusted and adjusted linear regression models. Adjusted models account for the following a priori defined covariates: Age, sex, body mass index, years of education, and income.

Abbreviations: B‐HCs, Black healthy controls; B‐PwMS, Black people with Multiple Sclerosis; CPL, Characteristic Path Length; DMN, Default mode network; FC, Functional Connectivity; GE, Global Efficiency; NWH‐HCs, Non‐Hispanic White healthy controls; NWH‐PwMS, Non‐Hispanic White people with Multiple Sclerosis; SC, Structural Connectivity; SMN, Sensorimotor network.

#### 3.1.1 Connector and Provincial Hubs

**FIGURE 2:**
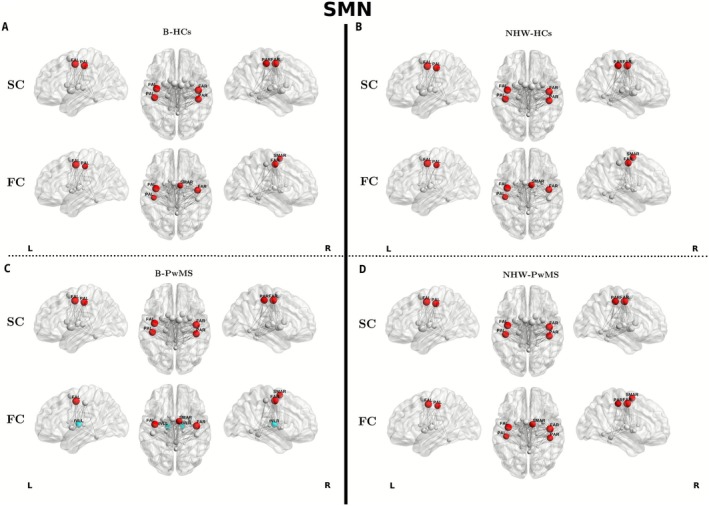
Provincial (red) and connector (light blue) hubs in the SMN for SC and FC in B‐HCs (A), NHW‐HCs (B), B‐PwMS (C), and NHW‐PwMS (D). B‐HCs, Black healthy controls; B‐PwMS, Black people with Multiple Sclerosis; FAL, left precentral gyrus; FAR, right precentral gyrus; FC, functional connectivity; NWH‐HCs, Non‐Hispanic White healthy controls; NWH‐PwMS, Non‐Hispanic White people with Multiple Sclerosis; PAL, left postcentral; PAR, right postcentral; SC, structural connectivity; SMAR, right supplementary motor area; SMN, sensorimotor network; tVLL, left thalamus ventral lateral; tVLR, right thalamus ventral lateral.

**FIGURE 3:**
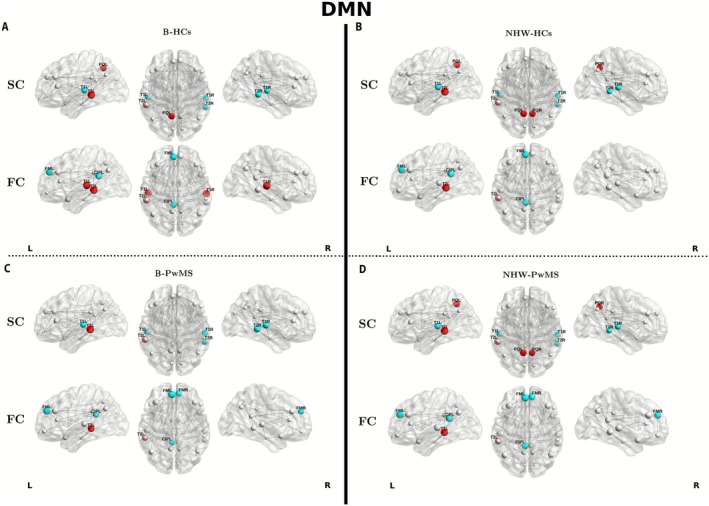
Provincial (red) and connector (light blue) hubs in the DMN for SC and FC in B‐HCs (A), NHW‐HCs (B), B‐PwMS (C), and NHW‐PwMS (D). B‐HCs, Black healthy controls; B‐PwMS, Black people with Multiple Sclerosis; CIPL, left cingulate posterior; DMN, default mode network; FC, functional connectivity; FML, left frontal superior medial; FMR, right frontal superior medial; NWH‐HCs, Non‐Hispanic White healthy controls; NWH‐PwMS, Non‐Hispanic White people with Multiple Sclerosis; PQL, left precuneus; PQR, right precuneus; SC, structural connectivity; T1L, left temporal superior; T1R, right temporal superior; T2L, left temporal middle; T2R, right temporal middle.

We found significant alterations in structural degree centrality values within the SMN and the DMN in both B‐PwMS and NHW‐PwMS (Table S3). We did not observe differences in SMN hub distribution between study subgroups; four provincial hubs and zero connector hubs were identified in all patients (Figure 2; Table S4). Regarding the DMN, B‐PwMS were missing the left precuneus as a provincial hub compared to B‐HCs, while no differences were noted in the NHW subgroups (Figure 3; Table S5).

### 3.2 Functional Connectivity Measures

No differences in global functional connectivity measures were observed among study subgroups (Table 2). No associations were found between SMN functional global efficiency and EDSS or between DMN functional global efficiency and SDMT.

#### 3.2.1 Connector and Provincial Hubs

We did not find differences in functional degree centrality values in the SMN nor the DMN between study subgroups. We observed a different distribution of connector and provincial hubs within the SMN in B‐PwMS compared to B‐HCs (Figure 2; Table S4). Specifically, B‐PwMS lost the left postcentral gyrus as a provincial hub and gained bilateral ventral lateral thalami as connector hubs. NHW‐PwMS retained the same provincial hubs as NHW‐HCs with the addition to the right postcentral gyrus. Regarding the DMN, B‐PwMS lost two provincial hubs and gained one connector hub compared to B‐HCs (Figure 3; Table S5), while NHW‐PwMS gained the right medial part of the superior frontal gyrus as a connector hub compared to NHW‐HCs.

### 3.3 Structure–Function Decoupling

**FIGURE 4:**
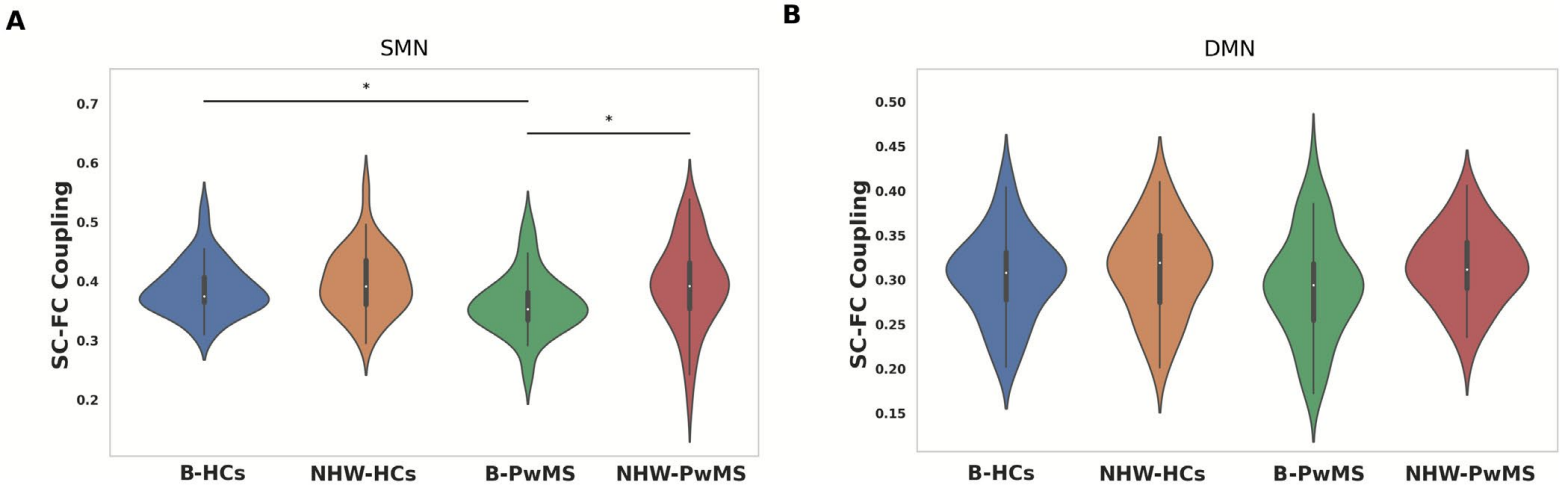
Structure–function coupling in the SMN (A) and DMN (B). B‐HCs, Black healthy controls; B‐PwMS, Black people with Multiple Sclerosis; DMN, default mode network; NWH‐PwMS, Non‐Hispanic white people with multiple sclerosis; NWH‐HCs, Non‐Hispanic White healthy controls; SMN, sensorimotor network.

For both HCs and PwMS, we found a significant correlation between structural and functional connectivity (*ρ* ranging from 0.12 to 0.61, all *p* < 10^−8^). B‐PwMS showed a higher degree of structure–function decoupling in the SMN (Figure 4; Tables 2 and 3) compared to both B‐HCs and NHW‐PwMS. Additionally, we observed trend‐level associations between structure–function decoupling and clinical measures. Specifically, a trend towards a higher degree of decoupling in the DMN was associated with lower SDMT scores (*ρ* = −0.20, *p* = 0.05), and in the SMN a trend towards higher decoupling was related to higher EDSS scores (*ρ* = 0.20, *p* = 0.06).

## 4 Discussion

In this study, we compared B‐PwMS and NHW‐PwMS with their respective healthy control groups and assessed potential differences in global structural and functional brain organization using connectomic analysis. Clinically, despite similar disease duration, treatment exposure, lesion load, and brain volumes, B‐PwMS showed higher EDSS scores and lower SDMT scores compared to NHW‐PwMS. However, these clinical differences did not remain significant after adjusting for age, sex, BMI, and SDoH.

As expected, both B‐PwMS and NHW‐PwMS exhibited reduced structural global efficiency and streamline density compared to their healthy counterparts. However, B‐PwMS showed more pronounced alterations in structural connectivity—particularly in degree centrality and hub distribution—than NHW‐PwMS, despite comparable disease metrics. Moreover, the characteristic path length within the SMN was increased in B‐PwMS relative to B‐HCs, suggesting significant disruption in network integration. These findings may reflect either differences in lesion topography—given the reported higher vulnerability in the brainstem, cerebellum, and spinal cord in B‐PwMS [4, 32, 33]—or a greater susceptibility to microstructural damage compared to NHW‐PwMS [13].

From a functional perspective, a similar pattern emerged: both B‐PwMS and NHW‐PwMS exhibited widespread redistribution of connector and provincial hubs within the SMN and DMN, with B‐PwMS showing more pronounced alterations. While this may indicate disease‐related neurofunctional reorganization, in which key hubs become overloaded and less efficient, we acknowledge that such changes could also reflect maladaptive processes, including microstructural damage or decreased network efficiency. Therefore, both adaptive and maladaptive interpretations should be considered. Longitudinal studies are needed to confirm whether the observed structural and functional alterations translate into meaningful differences in cognitive or physical outcomes.

The integration of structural and functional data has been previously pursued by either exploring the coupling between the two or, more recently, modeling the brain as a multilayer network [34]. Here, to integrate and deepen the insights provided by structural and functional connectivity data, we focused on the degree of structure–function coupling (or lack thereof) among a diverse cohort of PwMS. We observed a decoupling between structure and function in the SMN, with B‐PwMS showing significantly greater decoupling compared to both NHW‐PwMS and B‐HCs, and identified trends suggesting possible associations between higher structure–function decoupling in the SMN and DMN with worse physical and cognitive performances, respectively. Our results are in line with those found in longitudinal analyses of patients with a clinically isolated syndrome (CIS) suggestive of MS, which have shown preserved coupling compared to HCs at baseline, with subsequent structure–function decoupling over time [31]. These findings are also consistent with those of previous studies examining ischemic stroke patients with predominant subcortical involvement, where structure–function decoupling was related to the extent of motor impairment [35]. Similarly, network analysis in MS patients has identified an association between increased structure–function coupling and better cognitive performances, though only for long‐range connections [36, 37].

Changes in structure–function coupling, and consequent impact seem to vary not only according to the disease stage and topography of damage, but also in relation to specific features of the examined population. Indeed, individual differences in genetics, age, biological sex, health status, and environment affect the strength of structure–function coupling [35]. This is particularly relevant when investigating minority populations, as functional connectivity and structural integrity seem to be affected by environmental factors such as racial discrimination and socioeconomic status [6, 38, 39]. It is possible to speculate that B‐PwMS, having more neuron‐binding antibodies, and a higher IgG and eventually more gray matter damage [9, 10, 11], experience more synaptic and glial damage than NWH‐PwMS, with the result of a greater disconnection between structural capacity and functional output.

The cross‐sectional nature of our research precludes the establishment of a causal or temporal relationship, ultimately limiting our ability to discern the directionality of the observed relationships between disease impact, neural connectivity, and racial disparities. Second, although we took white matter lesions into account when reconstructing structural connectivity, there is still no clear consensus on the best way to incorporate lesion information into tractography. As a result, our findings may still be affected by the limitations of current methods in capturing lesion‐induced disconnection. Third, regarding cognitive assessment, reliance solely on the SDMT provides a limited perspective on cognitive functioning. Finally, although we included total income and years of education as surrogate measures of SDoH, these variables capture only a limited aspect of their multidimensional nature [40]. As a result, adjustment for SDoH in this study was necessarily limited. Moreover, socioeconomic status and race are complex constructs shaped by historical, societal, demographic, and behavioral factors, yet the causal pathways and underlying mechanisms were not directly examined here. Future studies could improve the interpretability and actionability of these findings by disentangling the contributions of individual variables and identifying upstream, downstream, or proximal factors and their interdependencies [41].

In conclusion, the results of this study illustrate that neural connectivity in PwMS is influenced by a combination of biological and sociodemographic factors. The more severe disruption in connectivity observed in B‐PwMS suggests that this population may require more aggressive or tailored comprehensive treatments to prevent disability accrual.

## Author Contributions

E.C. and G.B. contributed to the study design, data analysis, and interpretation of data. M.P. contributed to the interpretation of data. M.Po. and P.B. contributed to the analyses of data. N.G., C.W., C.R., J.H., and S.K. contributed to data acquisition. M.I. and F.L. supervised the study and contributed to the study design.

## Funding

This work was supported by the National Institutes of Health, 5R01NS100811 and Fondo Italiano per la Scienza, FIS_00002258.

## Conflicts of Interest

G.B. received personal fees from Novartis, Roche, Merck. F.L. reports grants and personal fees from Biogen, EMD Serono, Novartis, Sanofi/Genzyme, Roche/Genentech, Horizon Therapeutics/Amgen, BMS, Brainstorm Cell Therapeutics, Mylan/Viatris, Immunic, Avotres, LabCorp, Neuralight, SetPoint Medical, Hexal/Sandoz, Baim Institute, Sudo Biosciences, Lapix Therapeutics, Biohaven Pharmaceuticals, Abata Therapeutics, Cognito Therapeutics, ImmPACT Bio, InnoCare Pharma, Appia Bio. M.I. received grants from NIH, NMSS, FISM; received fees for consultation from BMS, Janssen, Roche, Genzyme, Merck, Biogen, and Novartis. All other authors declare no conflicts of interest.

## Supporting information


**Table S1:** Brain areas based on the AAL3 atlas that compose the SMN and DMN.
**Table S2:** Correlation matrix between MRI metrics, clinical variables, and social determinants of health in patients with multiple sclerosis.
**Table S3:** Brain regions showing significantly higher or lower degree centrality between‐group comparisons, based on structural connectivity networks.
**Table S4:** Connector and provincial hubs distribution in the SMN.
**Table S5:** Connector and provincial hubs distribution in the DMN.

## Data Availability

The data that support the findings of this study are available from the corresponding author upon reasonable request.
