# Supplementary material for: Structure–Function Decoupling of the Sensorimotor and Default Mode Networks in Black Americans With MS
Source: Ann Clin Transl Neurol. 2026 Feb 12:10.1002/acn3.70331. Online ahead of print. doi: 10.1002/acn3.70331 (PMC13394703; doi:10.1002/acn3.70331)
Supplement: Supplementary file 1 — Table S1: Brain areas based on the AAL3 atlas that compose the SMN and DMN. Table S2: Correlation matrix between MRI metrics, clinical variables, and social determinants of health in patients with multiple sclerosis. Table S3: Brain regions showing significantly higher or lower degree centrality between‐group comparisons, based on structural connectivity networks. Table S4: Connector and provincial hubs distribution in the SMN. Table S5: Connector and provincial hubs distribution in the DMN. [file ACN3-9999-0-s001.docx]

**Supplementary Materials**

**Supplementary Tables**

**Table S1. Brain areas based on the AAL3 atlas that compose the SMN and DMN.**

| **SMN** | | **DMN** | |
| --- | --- | --- | --- |
| **Brain areas** | **Abbreviation** | **Brain areas** | **Abbreviation** |
| Left Precentral gyrus  Right Precentral gyrus  Left Supplementary Motor Area  Right Supplementary Motor Area  Left Postcentral  Right Postcentral  Left Caudate  Right Caudate  Left Putamen  Right Putamen  Left Pallidum  Right Pallidum  Left Cerebellum 4_5  Right Cerebellum 4_5  Vermis 1_2  Vermis 3  Vermis 4_5  Left Thalamus LP  Right Thalamus LP  Left Thalamus VA  Right Thalamus VA  Left Thalamus VL  Right Thalamus VL  Left Thalamus VPL  Right Thalamus VPL | FAL  FAR  SMAL  SMAR  PAL  PAR  NCL  NCR  NLL  NLR  PALLL  PALLR  CER4_5L  CER4_5R  VER1_2  VER3  VER_4_5  tLPL  tLPR  tVAL  tVAR  tVLL  tVLR  tVPLL  tVPLR | Left Frontal Mid_2  Right Frontal Mid_2  Left Frontal Inf_Tri 2  Right Frontal Inf_Tri 2  Left Frontal Sup Medial  Right Frontal Sup Medial  Left Frontal Med Orb  Right Frontal Med Orb  Left Cingulate Post  Right Cingulate Post  Left ParaHippocampal  Right ParaHippocampal  Left Cuneus  Right Cuneus  Left SupraMarginal  Right SupraMarginal  Left Angular  Right Angular  Left Precuneus  Right Precuneus  Left Temporal Sup  Right Temporal Sup  Left Temporal Pole Sup  Right Temporal Pole Sup  Left Temporal Mid  Right Temporal Mid | F2_2L  F2_2R  F3TL  F3TR  FML  FMR  FMOL  FMOR  CIPL  CIPR  PHILPL  PHIPR  QL  QR  GSML  GSMR  GAL  GAR  PQL  PQR  T1L  T1R  T1AL  T1AR  T2L  T2R |

SMN = Sensorimotor network; DMN = Default mode network.

**Table S2. Correlation matrix between MRI metrics, clinical variables, and social determinants of health in patients with multiple sclerosis.**

|  | **Normalized total brain volume** | **Normalized cortical GM volume** | **Normalized WM volume** | **FLAIR lesion volume** | **EDSS** | **SDMT** |
| --- | --- | --- | --- | --- | --- | --- |
| Total income | **ρ =0.3**  **p=0.009** | **ρ =0.2**  **p=0.04** | **ρ =0.2**  **p=0.02** | ρ =-0.1  p=0.4 | **ρ =-0.3**  **p<0.001** | **ρ =0.4**  **p<0.001** |
| Education | ρ =0.2  p=0.08 | ρ =0.06  p=0.6 | **ρ =-0.2**  **p=0.006** | ρ =-0.06  p=0.6 | ρ =0.007  p=0.9 | ρ =0.2  p=0.06 |
| Body mass index | **ρ =-0.2**  **p=0.04** | ρ =-0.2  p=0.09 | **ρ =0.2**  **p=0.004** | ρ =0.08  p=0.4 | ρ =0.2  p=0.08 | ρ =-0.09  p=0.4 |

GM = Gray matter; WM= White Matter; EDSS= Expanded-Disability-Status-Scale; SDMT = Symbol-Digit-Modalities-Test.

**Table S3. Brain regions showing significantly higher or lower degree centrality between-group comparisons, based on structural connectivity networks.**

|  | **B-PwMS > B-HCs** | **NHW-PwMS > NHW -HCs** | **B-PwMS > NHW-PwMS** |
| --- | --- | --- | --- |
| Higher degree centrality value | Right caudate (SMN) | - | Left postcentral gyrus (SMN)  Left ventral lateral thalamus (SMN) |
| Lower degree centrality value | Left posterior lateral thalamus (SMN)  Left precuneus (DMN) | Left postcentral gyrus (SMN) | Left precuneus (DMN) |

B-HCs = Black healthy controls; B-PwMS = Black people with Multiple Sclerosis; NWH-HCs= Non-Hispanic White healthy controls; NWH-PwMS = Non-Hispanic White people with Multiple Sclerosis; SMN = Sensorimotor network; DMN = Default mode network.

**Table S4. Connector and provincial hubs distribution in the SMN**.

| **Groups** | **Provincial hubs** | | **Connector hubs** | |
| --- | --- | --- | --- | --- |
|  | **Structural** | **Functional** | **Structural** | **Functional** |
| B-HCs | Left precentral gyrus  Right precentral gyrus  Left postcentral gyrus  Right postcentral gyrus | Left precentral gyrus  Right precentral gyrus  Right supplementary motor area  Left postcentral gyrus |  |  |
| B-PwMS | Left precentral gyrus  Right precentral gyrus  Left postcentral gyrus  Right postcentral gyrus | Left precentral gyrus  Right precentral gyrus  Right supplementary motor area |  | Left ventral lateral thalamus  Right ventral lateral thalamus |
| NHW-HCs | Left precentral gyrus  Right precentral gyrus  Left postcentral gyrus  Right postcentral gyrus | Left precentral gyrus  Right precentral gyrus  Right supplementary motor area  Left postcentral gyrus |  |  |
| NHW-PwMS | Left precentral gyrus  Right precentral gyrus  Left postcentral gyrus  Right postcentral gyrus | Left precentral gyrus  Right precentral gyrus  Right supplementary motor area  Left postcentral gyrus  Right postcentral gyrus |  |  |

SMN = Sensorimotor network; B-HCs = Black healthy controls; B-PwMS = Black people with Multiple Sclerosis; NWH-HCs= Non-Hispanic White healthy controls; NWH- PwMS = Non-Hispanic White people with Multiple Sclerosis.

**Table S5. Connector and provincial hubs distribution in the DMN**.

| **Groups** | **Provincial hubs** | | **Connector hubs** | |
| --- | --- | --- | --- | --- |
|  | **Structural** | **Functional** | **Structural** | **Functional** |
| B-HCs | Left precuneus  Left middle temporal gyrus | Left superior frontal gyrus  Right superior frontal gyrus  Left middle temporal gyrus | Left superior frontal gyrus  Right superior frontal gyrus  Right middle temporal gyrus | Left superior frontal gyrus, medial  Left posterior cingulate gyrus |
| B-PwMS | Left middle temporal gyrus | Left middle temporal gyrus | Left superior frontal gyrus  Right superior frontal gyrus  Right middle temporal gyrus | Left superior frontal gyrus, medial  Right superior frontal gyrus, medial  Left posterior cingulate gyrus |
| NHW-HCs | Left precuneus  Left middle temporal gyrus  Right precuneus | Left middle temporal gyrus | Left superior frontal gyrus  Right superior frontal gyrus  Right middle temporal gyrus | Left superior frontal gyrus, medial  Left posterior cingulate gyrus |
| NHW-PwMS | Left precuneus  Left middle temporal gyrus  Right precuneus | Left middle temporal gyrus | Left superior frontal gyrus  Right superior frontal gyrus  Right middle temporal gyrus | Left superior frontal gyrus, medial  Right superior frontal gyrus, medial  Left posterior cingulate gyrus |

DMN = Default mode network; B-HCs = Black healthy controls; B-PwMS = Black people with Multiple Sclerosis; NWH-HCs= Non-Hispanic White healthy controls; NWH-PwMS = Non-Hispanic White people with Multiple Sclerosis.
